# Epidemiological and Economic Burden of Dengue in Mexico: Data Analysis from 2010 to 2020

**DOI:** 10.4269/ajtmh.24-0777

**Published:** 2025-10-07

**Authors:** Victoria Pando-Robles, Julio Alvarez-Obregón, Guadalupe Díaz del Castillo-Flores, Jesús Felipe González-Roldán, Eric Saúl Raga-Sarabia, Cassandra González-Acosta, Jorge Fernando Méndez-Galván

**Affiliations:** ^1^ Centro de Investigación Sobre Enfermedades Infecciosas, Instituto Nacional de Salud Pública, Morelos, Mexico;; ^2^ Takeda Mexico, Mexico City, Mexico;; ^3^ Dirección de Salud Pública, Veracruz, Mexico;; ^4^ Sociedad Mexicana de Salud Pública, Mexico City, Mexico;; ^5^ Centro Estatal de Cancerología, Veracruz, Mexico;; ^6^ Servicios de Salud, Morelos, Mexico;; ^7^ Hospital Infantil de México Federico Gómez, Mexico City, Mexico

## Abstract

Dengue is the most prevalent arboviral disease worldwide. This study aimed to describe the epidemiological and economic burden of dengue in Mexico by identifying comorbidities and conditions associated with clinical outcomes. A retrospective analysis of dengue epidemiological data in Mexico from 2010 to 2020 was conducted using surveillance system databases (Sistema Nacional de Vigilancia Epidemiológica and Sistema Único de Información para la Vigilancia Epidemiológica). Data on probable, laboratory-confirmed, outpatient, and hospitalized cases as well as deaths were collected along with comorbidities and pregnancy status; a survey was conducted to gather information on health care resources utilization. Sistema Nacional de Vigilancia Epidemiológica recorded 1,620,872 cases, among which there were 336,991 laboratory-confirmed cases, 110,437 hospitalizations, and 1,385 deaths. The hospitalization fatality rate increased from 0.72% to 2.6%. Age distribution of severe dengue shifted from predominantly affecting individuals 10–24 years old (2010–2016) to those 0–19 years old (2017–2020). These findings highlight the need for age-specific health care interventions. Comorbidities, such as diabetes, liver cirrhosis, hypertension, kidney disease, and hematological disorders, and pregnancy were significantly associated with an increased risk of hospitalization and mortality. Additionally, peptic ulcer was associated with a higher risk of hospitalization. The estimated annual medical cost during outbreak was U.S. $111,851,376 (2019, 211.0 [case] incidence) in contrast to pre-/postoutbreak, with total costs of U.S. $23,713,589 (2018, 62.4 [case] incidence) and U.S. $39,780,809 (2020, 94.1 [case] incidence), respectively. The robustness of collected data contributes to more comprehensive understanding of the public health implications of dengue, particularly during outbreaks.

## INTRODUCTION

Dengue fever is the most prevalent arboviral disease worldwide (40% of the human regions). Dengue is reported in 129 countries, with an estimated 390 million new infections occurring annually,^1^ including 96 million clinical cases, 500,000 severe dengue cases, and 22,000 deaths attributed to dengue, predominantly in children younger than 5 years old.^1^^,^^2^ The incidence of dengue has surged dramatically in recent decades, imposing a significant burden on health care systems and economies. Factors contributing to this rise include global trade, international travel, rapid urbanization, climate change, vector adaptation, insecticide resistance, the lack of effective vaccines, and budget-constrained vector control strategies.^3^

There are four serotypes of dengue virus (DENV; DENV-1, DENV-2, DENV-3, and DENV-4), which are transmitted to humans through the bites of female *Aedes* mosquitoes, primarily *Aedes aegypti* and *Aedes albopictus*.^4^ Approximately 75% of DENV infections are asymptomatic. However, when symptoms develop, typically 4–10 days after the bite, they range from mild febrile illness to life-threatening dengue shock syndrome.^3^^,^^5^ About 1 in 20 reported cases progresses to severe dengue, characterized by plasma leakage, severe hemorrhage, and organ failure, requiring intravenous fluid and supportive care.^6^ The postacute consequences of DENV infection impact productivity, health care costs, and quality-adjusted life years.^7^

Individuals in endemic regions are frequently exposed to DENV throughout their lifetime. Infection with any DENV serotype confers lifelong immunity against the same serotype and temporary crossprotection against the other three. However, secondary infection with a different serotype is a well-established risk factor for severe dengue because of antibody-dependent enhancement of infection, where subneutralizing antibodies facilitate viral entry into immune cells through Fc receptors, increasing viral replication. Additionally, crossreactive nonneutralizing antibodies can worsen the disease through antibody-dependent cellular cytotoxicity, contributing to dengue-associated vascular pathology.^8^^,^^9^

Currently, no specific antiviral treatments or highly effective vaccines are available.^10^ Dengvaxia (Sanofi Pasteur, Neuville-sur-Saône, France), the only approved vaccine in Mexico, is recommended for individuals ages 9–45 years old in endemic areas, but it has not been included in public immunization programs. As a result, vector control remains the primary strategy of dengue prevention.^8^^,^^9^

Mexico is an endemic country for dengue, with fluctuating incidence and periodic outbreaks, particularly in southeastern states, the Pacific coast, and the Gulf of Mexico. Dengue surveillance is mandatory and coordinated by the General Directorate of Epidemiology through a network of health services and public health laboratories across Mexico’s 32 states.^11^ Despite intensified prevention and vector control efforts, reported cases increased over the time. On the other hand, Mexico’s passive surveillance system faces challenges such as underreporting, misdiagnosis, and variations in reporting rates by severity and epidemic status, complicating accurate assessments of dengue’s true burden.^12^

Although previous studies have assessed the disease and economic burden,^12^^–^^16^ its impact varies by time, region, and age group, highlighting the needs for continued research. This study analyzes dengue epidemiology in Mexico from January 2010 to December 2020, focusing on disease severity, deaths by age group, geographic origin of reported cases, and clinical outcomes. We also examined associations with comorbidities and other conditions, and we estimated both direct and indirect costs of care, comparing epidemic peaks with surrounding nonepidemic years.

## MATERIALS AND METHODS

### Study design and population.

This observational, retrospective, descriptive database study was performed using available dengue case data from the National Epidemiology Surveillance System (Sistema Nacional de Vigilancia Epidemiológica [SINAVE]).^17^ The dataset reported dengue cases from public and private sectors between January 2010 and December 2020, encompassing suspected, probable, and laboratory-confirmed dengue cases; outpatient cases; hospitalized cases; and deaths. Additionally, a representative sample was drawn from SINAVE’s subset in the Single Information System for Epidemiological Surveillance (Sistema Único de Información para la Vigilancia Epidemiológica [SUIVE]).^17^ This dataset was the result of a research protocol developed in the year 2021, and it provides the disease occurrence, distribution over time and place, affected populations, risk factors, and health outcomes during the decade of 2010–2020.

**Figure 1.**
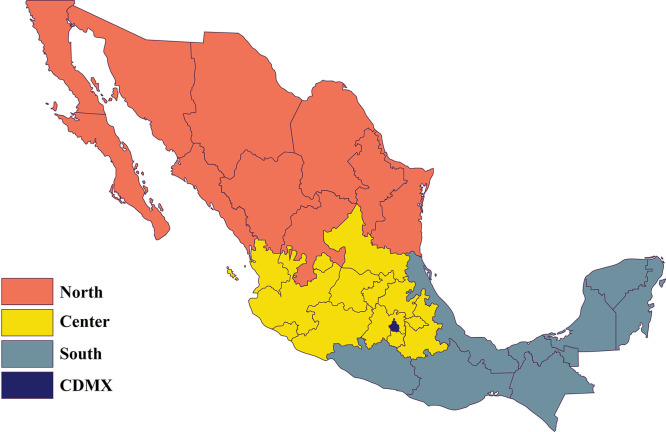
Four regions were defined considering geographical, climatological, and historical data of dengue incidence in Mexico for this study. This map is our own elaboration from the original public domain template, which is available at https://commons.wikimedia.org/wiki/File:Mexico_template.svg#/media/File:Mexico_template.svg. CDMX = Mexico City.

Four regions were defined, taking into account geographical, climatological, and historical data of dengue incidence: North (Baja California, Baja California Sur, Chihuahua, Coahuila, Durango, Nuevo León, Sonora, Sinaloa, Tamaulipas, and Zacatecas); Centre (Aguascalientes, Colima, Guanajuato, Hidalgo, Jalisco, Estado de México, Michoacán, Nayarit, Querétaro, San Luis Potosí, and Tlaxcala); South (Campeche, Chiapas, Guerrero, Morelos, Oaxaca, Puebla, Quintana Roo, Tabasco, Veracruz, and Yucatán); and Mexico City (where autochthonous transmission has not been reported). These regions are represented in Figure 1.

Dengue cases were defined according to the International Statistical Classification of Diseases and Related Health Problems, Tenth Revision (ICD-10), including all available aggregated data on probable cases, laboratory-confirmed cases, outpatient and hospitalized cases, and dengue-related deaths (Table 1). It should be noted that until 2016, cases were categorized as classical dengue, dengue with complications, dengue hemorrhagic fever, and dengue shock syndrome. In 2016, Mexico adopted the 2009 WHO dengue case classification.^6^

**Table 1:** Case definition of dengue and dengue-associated disease definition based on International Statistical Classification of Diseases and Related Health Problems, Tenth Revision codes

Disease Outcome	ICD-10 Code
Primary diagnosis	
Confirmed cases with dengue infection	
Dengue fever (classical dengue)	A90*
Dengue hemorrhagic fever	A91^†^
Dengue without warning signs	A97.0*
Dengue with warning signs	A97.1^†^
Severe dengue	A97.2^†^
Secondary or ancillary diagnosis	
Comorbidities in confirmed cases with dengue infection	
Diabetes mellitus	E10, E11, E12, E13, E14, O24
Hypertension	I10, I11, I12, I13, I15
CKD	N18, I12.0, E13
Chronic pulmonary disorders	J40–J99
Chronic liver disease	K70–K77
Blood disorders	D50–D89
Other hereditary disorders^‡^	D55–D56, E70–E88, Q00–Q99
Peptic ulcer disease and GE reflux	K27, K21.0
Pregnancy	O00–O30, O48, P01, Z32, Z34, Z35

CKD = chronic kidney disease; GE = gastroesophageal; ICD-10 = International Statistical Classification of Diseases and Related Health Problems, Tenth Revision.

* Nonsevere dengue includes nonsevere forms, such as dengue fever and dengue without warning signs.

^†^ Severe dengue includes dengue hemorrhagic fever, dengue with warning signs, and severe dengue.

^‡^ Other hereditary disorders include congenital heart disease, kidney disease, and metabolic disease among others.

For practical purposes, the data collected in this study were divided into two groups: 1) nonsevere dengue, which includes nonsevere forms such as dengue fever and dengue without warning signs, and 2) severe dengue, which includes dengue hemorrhagic fever, dengue with warning signs, and severe dengue. This classification aligns with the WHO’s revised 2009 criteria, which emphasize the inclusion of warning signs as diagnostic indicators for probable and potentially severe dengue.^18^ For baseline comorbidities and other conditions, such as pregnancy, primary diagnoses were initially identified and subsequently correlated with secondary and specific diagnoses.

Laboratory confirmation of the dengue diagnosis was validated in agreement with the National Network of Public Health Laboratories for Dengue and Other Arboviral Diseases surveillance’s operational procedures.^11^

### Data sources.

Population estimates were obtained from the National Institute of Statistical and Geography^19^ and the National Population Council.^20^ Data of dengue cases, outcomes, and comorbidities were obtained from SINAVE and SUIVE. The numbers of days of hospitalization for dengue cases were obtained from the Dynamic Cubes of the General Directorate of Health Information.^21^

As previously mentioned, the baseline comorbidities (including but not limited to diabetes, hemorrhagic disorders, hypertension, liver cirrhosis, chronic kidney disease, and peptic ulcer disease), and pregnancies were obtained from SINAVE. The population was divided into children (0–4 and 5–9 years of age [YoA]), adolescents (10–14 and 15–19 YoA), young adults (20–39 YoA), adults (40–59 YoA), and elderly (≥60 YoA).

### Costs.

In this work, we used the term “direct costs” to refer to the public sector medical cost. In turn, the “indirect costs” are those that are borne by the health beneficiary and are seen as an out-of-pocket expenditure. In this study, for the estimation of direct medical costs of patients with nonsevere and severe dengue with or without comorbidities, we considered the following costs: laboratory and office based, emergency room, and hospitalization (general or intensive care unit); medical surgical procedures; and medications (including transfusion of red blood cells and platelets, fresh plasma, and hemodialysis sessions) for management. Indirect costs included the average number of paid sick days (only for the public sector) among the adult population. This work is limited to analyzing data *in silico*; thus, the protocol that sustains this research does not contemplate the out-of-pocket payments by patients’ families, which would imply an amount of field work.

Direct medical costs were estimated from a Delphi expert panel survey^22^ obtaining answers from all kinds of institutions in Mexico and the Instituto Mexicano del Seguro Social (IMSS) unit costs by level of medical care (Licitación Pública Internacional ITB/2020/17938); specific cost tabulators for private sector institutions were used considering that IMSS is the biggest public institution in Mexico and provides standardized information regarding cost. In the first round, the panelists answered an electronic survey. The responses were analyzed to determine the minimum, median, and maximum points. In the second round, panelists were invited to discuss the answers at extreme points (minimum or maximum) and to reconsider their responses until a consensus was reached on each question. This process allowed us to obtain rates of resource consumption linked to the medical care of dengue patients. Based on the panel’s consumption rates and relevant sources providing unit costs for medical care and treatment expenses, we were able to ascertain the per capita cost and total cost of medical care at the national level.

To estimate indirect medical costs and determine the average daily income, reports from Comisión Nacional de Salarios Mínimos (Secretaria del Trabajo y Previsión-Labor Ministry) were used.^23^ Days of paid sick leave because of dengue were estimated by taking into account data published by Zeng et al.,^24^ in which the average duration of illness was 13.1 days for ambulatory cases; this was used as a proxy for an ambulatory case with no comorbidities. The proportion of patients of working age was estimated from the age distribution obtained from the data obtained from the SUIVE database and imputed to the SINAVE population to obtain the national size of the indirect costs. These days were multiplied by the cost of an average labor day income at U.S. $19.0. To convert Mexican pesos into U.S. dollars, an exchange rate of $20.0 pesos per dollar was considered.

## STATISTICAL ANALYSES

Using the SINAVE/SUIVE data, descriptive statistics were provided for probable, laboratory-confirmed, outpatient, and hospitalized cases as well as the occurrence of death because of dengue. The data collected for this study were used to identify variables related to dengue severity, year, age or age group, sex, and region. To estimate health care resource utilization, a data collection survey was uploaded to an interactive platform to collect information from a survey delivered to a panel of experts, which consisted of 14 specialists in infectious diseases and pediatrics from public (8 specialists) and private (6 specialists) health care sectors in Mexico as described in the previous section.

To analyze the updated cost impact in recent years, when a massive dengue epidemic was observed during the analysis period, 3 years were analyzed: 2018 (low epidemic), 2019 (major epidemic), and 2020 (low epidemic). Eight dengue-related health conditions were analyzed for health care costs: 1) outpatient cases with nonsevere dengue without comorbidities, 2) outpatient cases with severe dengue without comorbidities, 3) outpatient cases with nonsevere dengue with comorbidities, 4) outpatient cases with severe dengue with comorbidities, 5) hospitalized cases with nonsevere dengue without comorbidities, 6) hospitalized cases with severe dengue without comorbidities, 7) hospitalized cases with nonsevere dengue with comorbidities, and 8) hospitalized cases with severe dengue with comorbidities. Cost data are presented in U.S. dollars. For all outcomes, descriptive statistical analysis was performed to estimate the total number of occurrences of each outcome.

A univariate logistic regression model was used to estimate the hospitalization and mortality odds ratio (OR) as a function of dengue severity and comorbidities. The accumulated OR was calculated for the entire observation period of 10 years, comparing the individuals who had the condition (comorbidity) with those who did not. The following comorbidities/conditions were used to fit the model because they were associated with significant complications and were the most frequent: diabetes, blood disorders, hypertension, peptic ulcer disease, liver cirrhosis, chronic kidney disease, and pregnancy. To avoid calculation bias, only the registers with only one comorbidity were considered. In this study, age was not considered because comorbidities are more prevalent in the adult population. Each logistic regression outcome was reported with its respective OR for each variable and a 95% CI, and statistical significance was set at a *P*-value <0.05. The analysis was performed using Stata 15 statistical software (StataCorp, College Station, TX).

## RESULTS

### Epidemiology of dengue.

A total of 1,620,872 total reported cases of dengue were observed in the study period from 2010 to 2020 at the SINAVE system. According to the current diagnostic algorithm for dengue, 30% of probable cases during an outbreak should be evaluated by laboratory testing.^11^ From this, 336,991 (20.7%) were laboratory-confirmed cases, of which 226,554 (67.2%) received ambulatory care, and 110,437 (32.8%) were hospitalized; 1,385 (1.2%) of these patients died (see Table 2).

**Table 2:** Number of notified dengue cases, type of care, deaths, and population in Mexico from 2010 to 2020

Year	Total Reported Cases (SINAVE)*	Probable Cases (SUIVE)^†^	Laboratory-Confirmed Cases^†^				Total Population^‡^
			Total	Outpatient	Hospitalized		
					Total	Deaths	
2010	121,499	121,713	30,149	22,478	7,671	62	114,756,059
2011	69,910	21,847	16,583	9,758	6,825	50	116,446,180
2012	165,749	84,611	52,128	30,708	21,420	170	118,060,514
2013	231,498	125,795	63,984	40,335	23,649	192	119,597,654
2014	124,943	54,948	32,616	22,022	10,594	76	121,048,604
2015	219,593	67,336	27,175	20,300	6,875	95	122,368,490
2016	130,069	45,624	17,904	13,944	3,960	75	123,587,407
2017	89,893	38,343	14,490	11,241	3,249	65	124,777,172
2018	78,621	30,708	13,340	8,306	5,034	75	125,995,825
2019	268,458	157,018	43,396	28,194	15,202	371	127,215,666
2020	120,639	56,467	25,226	19,268	5,958	154	128,209,170
**2010–2020**	**1,620,872**	**804,410**	**336,991**	**226,554**	**110,437**	**1,385**	**122,005,704** ^§^

The last bolded line corresponds to the sum of analyzed cases, except for the last column, which shows the average population. SINAVE = National Epidemiology Surveillance System Database; SUIVE = Single Information System for Epidemiological Surveillance.

* Information was obtained from the epidemiological overview of dengue 2010–2020 from SINAVE.^21^

^†^ Information was obtained from the morbidity yearbooks 1984–2022 from SUIVE.^21^

^‡^ Adjusted population was according to the latest report from the National Population Council available at https://datos.gob.mx/dataset/proyecciones-de-poblacion (revised December 12, 2023).

^§^ Average annual population. Total reported cases included suspicious, probable, and laboratory-confirmed cases.

Considering the nationwide sample of 804,410 reported cases with the more complete data available in SUIVE, from the total sample, 87.2% (range: 70.6–92.4%) were reported as nonsevere dengue cases, and 12.8% (range: 7.6–29.4%) were reported as severe dengue cases (Supplemental Table 1). From the sample, 55.5% (range: 52.1–58.1%) of patients were female, and 44.5% (range: 41.6–47.9%) of patients were male (Supplemental Table 2). Moreover, from the laboratory-confirmed dengue cases set, 71.5% (range: 64.1–79.8%) of cases were diagnosed as nonsevere dengue, whereas 28.5% (range: 20.2–35.9%) of cases were diagnosed as severe (Supplemental Table 4). A complete listing of these results is provided in Supplemental Tables 1–22.

**Figure 2.**
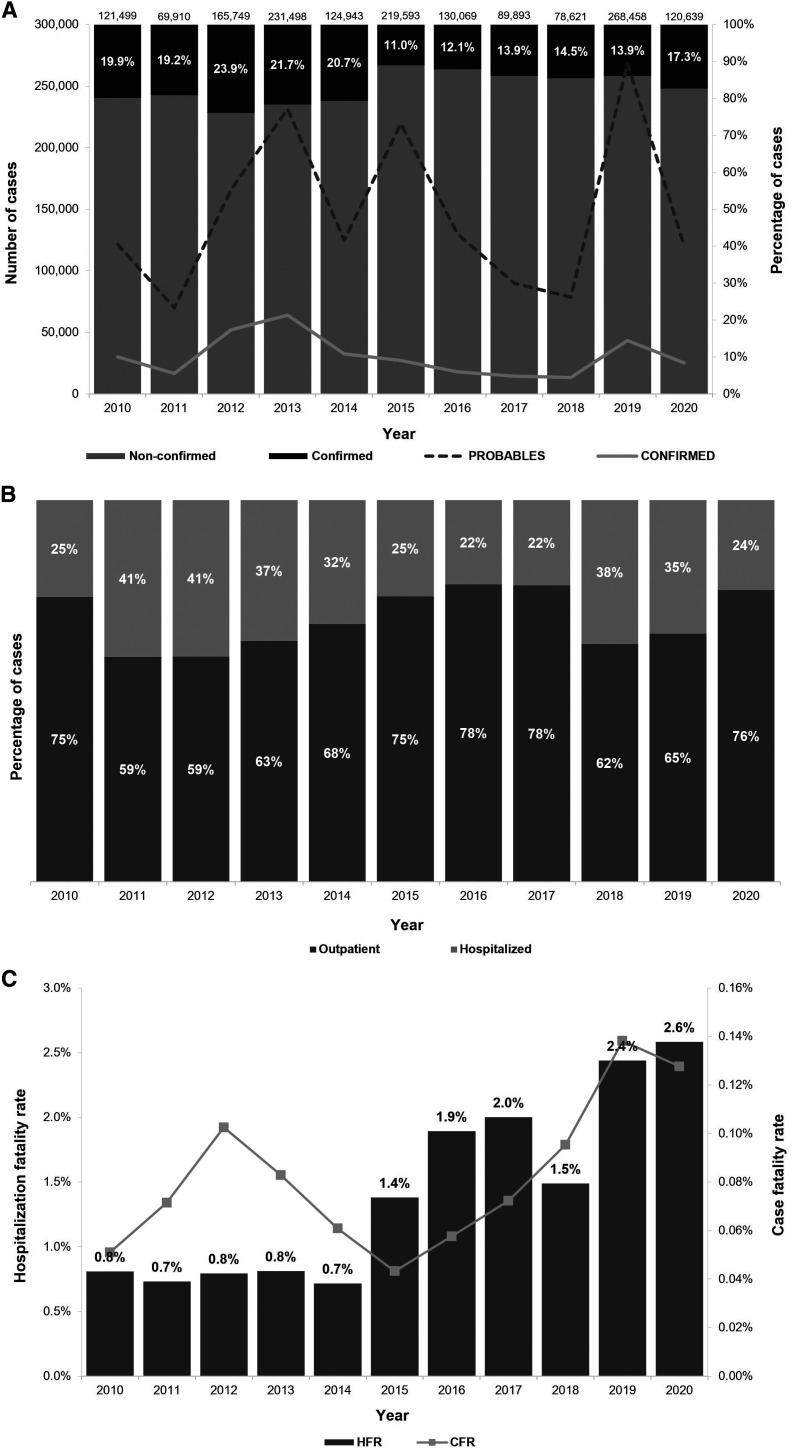
Epidemiologic profile of dengue in Mexico from 2010 to 2020. (**A**) Number and percentage of total and laboratory-confirmed dengue cases. (**B**) Percentage of laboratory-confirmed dengue cases considering the site of care. (**C**) Hospitalization fatality rate/case fatality rate (in percentage). All of these indicators were calculated from Table 2.

Dengue fever dynamics are known to be particularly complex, with large fluctuations in disease cases. Figure 2 shows the epidemiologic profile of dengue cases in Mexico from 2010 to 2020. The epidemic peaks of total reported dengue cases were recorded in 2013, 2015, and 2019, whereas the highest numbers of laboratory-confirmed dengue cases were recorded in 2012, 2013, and 2019.

In Mexico, the Health Secretary requests laboratory confirmation for at least 30% of all probable dengue cases.^11^ Our results showed an average of 17.1% (range: 11.0–23.9%) dengue confirmation, with a clear decrease in the percentage of laboratory-confirmed cases from 2015 to 2020 compared with previous years (Figure 2A). Figure 2B shows laboratory-confirmed dengue cases only. As mentioned above, 110,437 laboratory-confirmed dengue cases were hospitalized, representing 32.8% (range: 22.1–41.2%) of the total laboratory-confirmed dengue cases. Outpatient care represented 67.2% of laboratory-confirmed cases during the analyzed period. The highest hospitalization rates because of dengue occurred in 2011, 2012, and 2018 (Figure 2B). Despite the unchanged detection policy during the study period, the confirmation percentage shown in Figure 2A varies, indicating the limitations of the passive surveillance system.

The case fatality rate (CFR), defined as the number of dengue-related deaths divided by the total number of dengue cases, varies depending on infection severity and the presence of comorbidities. Between 2010 and 2020, a total of 1,385 dengue-related deaths were reported. During this period, the CFR ranged from 0.06% to 0.14%, whereas hospitalization fatality rate (HFR), which is defined as the number among hospitalized cases, averages 1.25% (range: 0.8–2.6%). The HFR remained below 1% between 2010 and 2014 but increased between 2015 and 2020 (Figure 2C). A total of 737 deaths occurred in females, accounting for a higher proportion of deaths than in males: 53.2% (range: 45.3–68.0%) compared with 46.8% (range: 32.0–54.7%). All reported deaths were from cases classified as severe dengue cases, with the majority occurring in hospitalized patients (1,295 [93.5%; range: 85.1–100%]) rather than outpatients cases (90 [6.5%; range: 0.0–14.9%]) (Supplemental Table 11). These finding indicate an overall increase in both CFR and HFR between 2010 and 2020.

**Figure 3.**
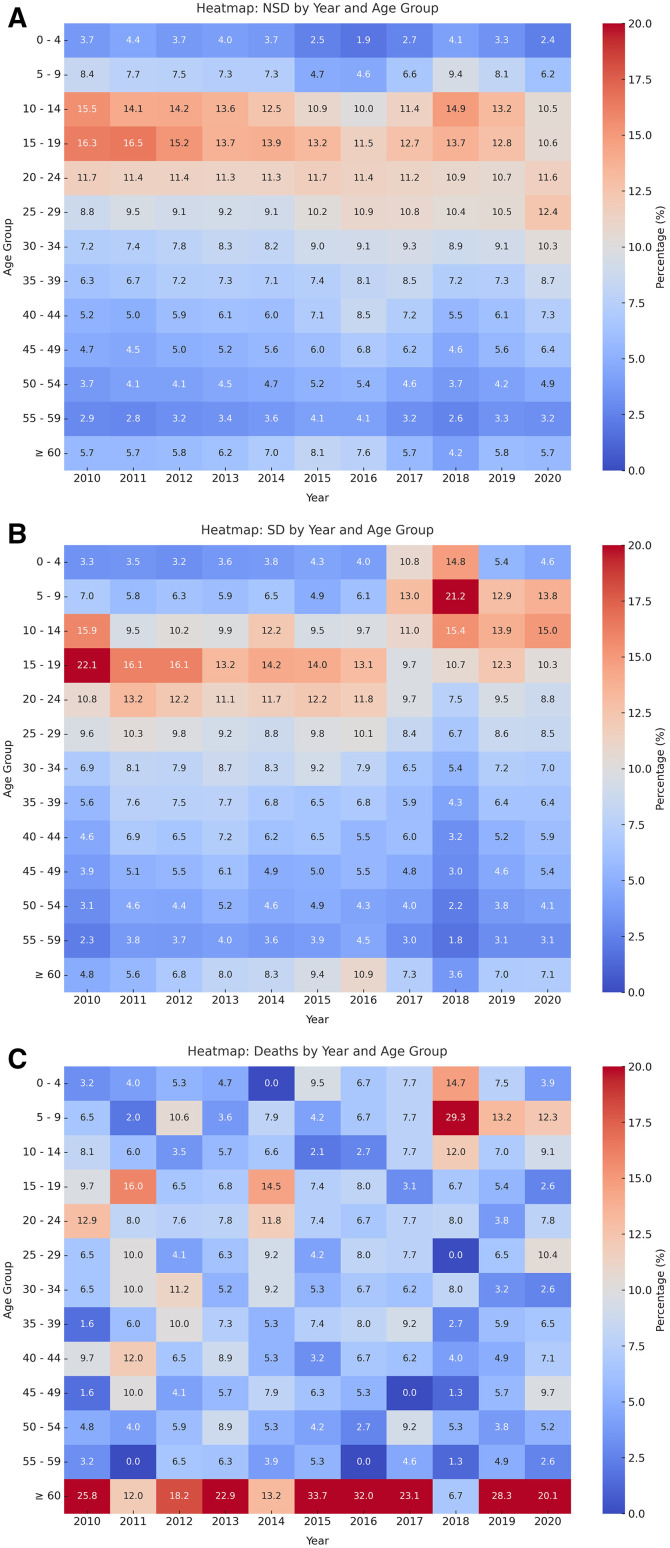
Distribution of dengue cases by year and age groups. The percentages of dengue cases and deaths are shown for each age group: (**A**) nonsevere dengue (NSD) cases, (**B**) severe dengue (SD) cases, and (**C**) deaths.

Dengue fever can affect individuals of all ages, but certain age groups are more vulnerable. Figure 3 illustrates the age-related variation in dengue epidemiology from 2010 to 2020. The temporal distribution of nonsevere dengue cases (Figure 3A) shows a higher prevalence among individuals younger than 30 YoA. In contrast, the distribution of severe cases (Figure 3B) reveals a shift in the most affected age groups; from 2010 to 2016, the highest prevalence occurred in the 10–24 YoA group (average of 38%), whereas from 2017 to 2020, it was concentrated among individuals younger than 19 YoA (48%), highlighting distinct epidemic dynamics.

Focusing specifically on severe dengue cases, the 5–9 YoA group exhibited the highest burden from 2017 to 2019, which accounted for 21.2% of cases in the year 2018, making it the most affected group. This was followed by the 10–14 YoA group (15.4%) and the 0–4 YoA group (14.8%) (Figure 3B).

Regarding mortality, the 5–9 YoA group experienced the highest proportion of deaths, peaking at 29.3% in 2018. Among adults, elderly patients (older than 60 YoA) were the most affected, with 32.0% of fatal cases in 2016 and another peak of 28.3% in 2019 (Figure 3C).

When visualizing the data as incidence rate per 100,000 inhabitants, the overall average incidence rate of laboratory-confirmed dengue cases from 2010 to 2020 was 25.3 (range: 10.6–56.9). The highest incidences during this period were observed in 2012, 2013, and 2019, reaching 44.2, 53.5, and 34.1, respectively (Table 3). Nonsevere dengue cases were reported with a higher incidence (18.1; range: 6.8–36.9) compared with severe dengue cases (7.2; range: 3.8–16.6). The regional breakdown was particularly noteworthy. The data indicated that the Centre region of Mexico has the lowest incidence, that the North region falls in the middle, and that the South region has the highest incidence when the Centre is used as a baseline. In comparison with the Centre, the North has roughly twice the incidence rate and half the HFR. The southern region had the highest incidence and HFRs compared with the other regions of the country; it had about three times the incidence rate and HFR compared with the Centre (Table 3), indicating that dengue burden disease in Mexico is concentrated in the states of Campeche, Chiapas, Guerrero, Morelos, Oaxaca, Puebla, Quintana Roo, Tabasco, Veracruz, and Yucatán.

**Table 3:** Incidence rate by severity and region for confirmed dengue cases and hospitalization fatality rate

Year	Total Reported Cases*	Laboratory-Confirmed Cases						Hospitalization Fatality Rate		
		Total Cases*	Severity*		Region^†^			Region^‡^		
			NSD	SD	North	Centre	South	North	Centre	South
2010	105.9	26.3	20.6	5.7	29.6	9.4	52.5	0.14	0.13	0.53
2011	60.0	14.2	9.9	4.3	3.8	2.6	41.3	0.09	0.00	0.64
2012	140.4	44.2	28.3	15.9	18.6	8.9	121.4	0.03	0.02	0.74
2013	193.6	53.5	36.9	16.6	62.5	24.8	97.3	0.08	0.15	0.58
2014	103.2	26.9	19.6	7.3	51.0	8.1	39.4	0.37	0.00	0.35
2015	179.5	22.2	17.6	4.6	23.7	15.5	35.3	0.26	0.25	0.87
2016	105.2	14.5	11.5	3.0	16.4	10.6	21.6	0.23	0.18	1.49
2017	72.0	11.6	9.3	2.3	11.7	14.0	11.2	0.34	0.62	1.05
2018	62.4	10.6	6.8	3.7	6.3	8.5	19.4	0.08	0.18	1.23
2019	211.0	34.1	22.7	11.4	7.8	34.8	62.8	0.04	0.84	1.56
2020	94.1	19.7	15.4	4.3	22.7	26.0	13.8	0.44	1.12	1.02
**2010–2020**	**120.7**	**25.3**	**18.1**	**7.2**	**23.1**	**14.8**	**46.9**	**0.19**	**0.32**	**0.92**

The last bolded line corresponds to the average results. NSD = nonsevere dengue; SD = severe dengue. Incidence rate is per 100,000 inhabitants. Information was sourced from the National Epidemiology Surveillance System Database/Single Information System for Epidemiological Surveillance.

* Estimated incidence rate from the total national population.

^†^ Estimated incidence rate from the total population by region.

^‡^ Estimated hospitalization fatality rate per 100 hospitalized cases.

### Influence of comorbidities on dengue clinical outcomes.

From 2011 to 2020 (no data were available for 2010), 8,731 laboratory-confirmed cases had at least one comorbidity at diagnosis, representing 2.6% (range: 1.9–3.8%) of the total cases. The distribution of cases by the number of concurrent comorbidities was as follows: one comorbidity in 6,271 cases (71.8% [range: 65.7–80.9%]), two comorbidities in 2,287 cases (26.2% [range: 18.1–31.0%]), three comorbidities in 153 cases (1.8% [range: 0.7–4.1%]), and four comorbidities in 20 cases (0.2% [range: 0.0–0.7%]) (Supplemental Table 12). The most frequent comorbidities selected for their association with the major complications included diabetes, hemorrhagic disorders, hypertension, liver diseases, and chronic kidney disease. Given its role as a risk factor for worse disease outcomes, pregnancy was distinguished as a unique condition in relation to dengue.

Diabetes and hypertension were the most reported comorbidities, affecting 4,501 and 2,434 cases, respectively, followed by hemorrhagic disorders (1,143 cases), peptic ulcer (239 cases), liver cirrhosis (180 cases), immunosuppression (122 cases), and chronic kidney disease (112 cases). Additionally, 5,517 pregnant women were diagnosed with dengue, representing 3.3% (range: 2.1–5.5%) of laboratory-confirmed cases among women from 2011 to 2020 (Supplemental Table 12).

### Association of comorbidities with hospitalization and death.

Secondary infections are widely recognized as a major risk factor for severe dengue.^8^ However, other conditions, like pregnancy and certain comorbidities, also influence disease severity. The analyzed database assesses the ORs of hospitalization, severe dengue, and death in relation to these factors (Table 4). These data underscore the need for vigilant monitoring and timely therapeutic interventions in patients with these risk factors.

**Table 4:** Odds ratios of hospitalization and death for different comorbidities and conditions

Condition (Rank)	OR	95% CI	*P*-Value	Attributable Fraction, *n* (%)*
Hospitalization OR (all cases)				
Peptic ulcer disease (5)	20.85	12.11–36.16	<0.001	215 (0.063)
Pregnancy (1)	15.25	13.95–16.71	<0.001	4,449 (1.320)
Liver cirrhosis (6)	8.72	5.06–16.59	<0.001	136 (0.040)
Diabetes (2)	8.68	7.76–9.72	<0.001	3,644 (0.580)
Hypertension (3)	7.84	6.68–9.22	<0.001	1,956 (0.022)
Kidney disease (7)	4.89	2.04–13.82	<0.001	76 (0.207)
Hemorrhagic disorders (4)	3.02	2.46–3.73	<0.001	699 (0.063)
Hospitalization OR (nonsevere cases)				
Peptic ulcer disease (5)	26.5	15.76–44.57	<0.001	68 (0.020)
Pregnancy (1)	18.98	17.62–20.44	<0.001	2,667 (0.791)
Diabetes (2)	9.78	8.89–10.76	<0.001	1,028 (0.305)
Liver cirrhosis (6)	9.24	6.15–13.87	<0.001	54 (0.016)
Hypertension (3)	8.34	7.32–9.50	<0.001	497 (0.147)
Kidney disease (7)	3.71	2.09–6.57	<0.001	18 (0.005)
Hemorrhagic disorders (4)	3.09	2.59–3.70	<0.001	174 (0.052)
Hospitalization OR (severe cases)				
Kidney disease (7)	7.85	1.91–32.16	0.004	58 (0.017)
Liver cirrhosis (6)	7.40	2.33–23.43	0.001	82 (0.024)
Peptic ulcer disease (5)	6.63	2.93–15.01	<0.001	147 (0.044)
Hypertension (3)	6.58	5.08–8.53	<0.001	1,459 (0.433)
Diabetes (2)	5.90	4.91–7.09	<0.001	2,616 (0.776)
Pregnancy (1)	5.88	4.71–7.34	<0.001	1,782 (0.529)
Hemorrhagic disorders (4)	2.84	2.12–3.8	<0.001	525 (0.156)
Deaths OR (severe cases)				
Hemorrhagic disorders (4)	5.95	4.29–8.25	<0.001	37 (0.011)
Kidney disease (7)	5.69	2.06–15.72	0.001	4 (0.001)
Liver cirrhosis (6)	4.98	2.01–12.31	0.001	5 (0.001)
Diabetes (2)	3.68	3.04–4.46	<0.001	120 (0.036)
Hypertension (3)	3.21	2.46–4.20	<0.001	59 (0.018)
Peptic ulcer disease (5)	2.13	0.79–5.78	0.134	4 (0.001)
Pregnancy (1)	2.1	1.57–8.82	<0.001	47 (0.014)

OR = odds ratio. Only statistically significant results are included (source: National Epidemiology Surveillance System Database confirmed cases).

* The total number of all cases is used as the denominator.

Table 4 shows that the OR of hospitalization for nonsevere dengue patients was significantly elevated with comorbidities. Peptic ulcer disease showed the highest OR (26.5) for hospitalization followed by pregnancy (OR: 18.9). Patients with diabetes, liver cirrhosis, and hypertension had approximately nine times higher odds of developing severe dengue than those without these comorbidities. Renal disease, liver cirrhosis, peptic ulcer disease, and hypertension emerged as the most critical comorbidities associated with higher hospitalization risk in severe dengue cases. Given the high prevalence of diabetes mellitus and arterial hypertension in Mexico, further research is warranted to explore their impact on dengue progression, especially among age groups prone to severe dengue.

Our findings indicate a strong association between certain comorbidities and the OR of death in severe dengue cases. Notably, this association was observed in cases affected by severe dengue. Among the comorbidities studied, peptic ulcer disease was the only one without a significant link to mortality. Table 4 shows that blood disorders, kidney disease, and liver cirrhosis are associated with an increased OR of death in severe dengue cases. To avoid interpretation bias, only the registers with only one comorbidity were considered for the calculation.

It is important to consider that the cumulated OR calculated over long periods can be influenced by variability in conditions, such as epidemiological transitions or changes in health policies during government transitions, which may impact the results. Additionally, the wide CIs observed around the OR appear to correlate with the number of comorbidity cases, indicating that these broad intervals are likely because of the effect of low prevalence and small sample size. Regarding higher OR values, we emphasized that OR describes the impact of dengue infection in persons with comorbidities, and it does not represent the most frequent comorbidity observed in the database.

### Economic component.

#### Direct medical costs.

Considering all of the probable cases, which include both laboratory-confirmed and nonconfirmed cases that received treatment, the average total medical care costs were U.S. $60,713,369 (U.S. $772.2 per patient) in the preoutbreak period (2018), U.S. $191,235,380 (U.S. $712.3 per patient) during the outbreak period (2019), and U.S. $84,989,764 (U.S. $704.5 per patient) in the postoutbreak period (2020) (Supplemental Table 20). During an outbreak year, like 2019, total cost can increase by up to 79% compared with an endemic transition year, such as 2018. The cost per patient varies with the severity of cases observed rather than the number of cases.

Regarding the cost of dengue by severity, for nonsevere dengue cases, cumulative expenses accounted for 43.9% of the total in the preoutbreak year (2018), rising to 70.2% during the outbreak (2019) and 66.1% in the postoutbreak year (2020). Conversely, in a year with normal dengue transmission (2018), severe dengue represented 44% of the total cost. In 2018, the highest cost burden was among children and teenagers ages 5–19 YoA, who account for 43.1% of total costs. In 2019 and 2020, patients ages 5–29 YoA represented 56.7% and 53.2% of the total costs, respectively.

#### Indirect medical costs.

Indirect medical costs were calculated based on the average daily income (U.S. $19.0) multiplied by the average days of paid sick leave. Although most dengue cases occur in children who do not contribute to these costs, the following averages represent the cumulated indirect costs for all cases. Further details on cost assignment are in Supplemental Tables 1–22.

In 2018, nonsevere dengue cases averaged 13.5 days of incapacity, resulting in a total cost of U.S. $641,193 (U.S. $24.8 per patient) across outpatients and inpatients with and without comorbidities. Severe dengue cases had an average of 14.9 incapacity days, with a total cost of U.S. $95,165 (U.S. $20.4 per patient). For all severities, the total national cost was U.S. $736,359 (U.S. $24.2 per patient).

In 2019, nonsevere dengue cases had an average of 13.6 incapacity days, totaling U.S. $3,555,818 (U.S. $25.1 per patient), whereas severe dengue cases averaged 22.1 days, costing U.S. $517,295 (U.S. $35.8 per patient). The national cost for all severities was U.S. $4,073,114 (U.S. $26.1 per patient).

Finally, in 2020, nonsevere dengue cases resulted in an average of 13.4 days of incapacity, with a total cost of U.S. $2,130,837 ( U.S. $42.4 per patient). Severe cases averaged incapacity duration of 22.2 days, with a total cost of U.S. $108,950 (U.S. $19.9 per patient). For all severities, the national cost was U.S. $2,239,787 (U.S. $40.2 per patient).

## DISCUSSION

Dengue is consistently present in Mexico; 60% of the national territory presents ideal conditions for the development of mosquito vectors,^25^ where seasonal outbreaks are common, typically peaking during the rainy season when mosquito populations increase. Local spatial variations in DENV transmission are strongly influenced by factors such as rainfall; temperature; unplanned urbanization; climate change; absence of reliable piped water; ineffective vector control strategies; and population mobility because of work, migration, or international travel.^1^^,^^2^ This study provides a longitudinal retrospective analysis of the dengue burden in Mexico from 2010 to 2020, estimating the economic costs associated with dengue management and examining the comorbidities linked to severe dengue cases.

Dengue cases are recorded through passive surveillance, and only a fraction of patients with undifferentiated fever are tested. Studies have estimated significant underreporting of symptomatic dengue cases. Sarti et al.^26^ estimated an underreporting rate of 8.4 times, and Martinez-Vega et al.^27^ found 33.3% underreported cases and 68.2% undernotified cases for Mexico; however, considering the differences in epidemiological vigilance,^28^ a direct comparison could be complex as Sarti et al.^26^ showed for Latin American countries.^21^ Additionally, dengue seroprevalence in endemic states, such as Morelos and Yucatan, was approximately 75%, suggesting widespread exposure to the virus.^29^^,^^30^ Overall, the true burden of dengue is likely underestimated in surveillance data because of high rates of asymptomatic infections, self-management, misdiagnosis, and especially, incomplete reporting from private health care facilities. Accurate dengue reporting is essential for public health officials to understand disease burden and assess the cost-effectiveness of interventions, but it remains challenging because of the fluctuations on incidence through time and states inside the country.^13^

Consistent with the findings reported by Torres-Galicia et al.,^31^ our data indicated that dengue incidence is highest among individuals younger than 30 years old. Shifts in the population at risk, especially among those ages 15 and 19 YoA, have been accompanied by increased severity in dengue outcomes. Notably, severe cases and hospitalizations in the 0–19 YoA group have risen since 2017. Similar trends have been observed in Oceania, South Asia, and Southeast Asia, where the highest incidence is seen in the 5–14 YoA group.^32^ In Latin America, Brazil reported the highest number of cases in the 20–39 YoA group.^33^ Conversely, dengue-related deaths are most common in adults older than 60 YoA. However, since 2018, the proportion of deaths among those younger than 19 YoA has increased 2.5-fold compared with previous years, a trend also observed by Macias et al.^34^ in the Mexican population. These data are significant when considering health impact in terms of disability-adjusted life years (DALYs); the global burden of dengue was estimated at 2.28 million DALYs in 2016,^15^ with the most affected groups being young children (1–4 YoA) and adults (25–44 YoA). An increase of burden up to 2.38 million DALY was estimated in 2019.^35^

Our data indicate that 17.1% of cases (336,991 patients) were confirmed through laboratory testing, with 31.2% of cases (110,437 patients) resulting in hospitalization and 28.5% of cases (96,037 patients) classified as severe dengue cases. These findings are consistent with data from the Pan American Health Organization, which reported that Mexico accounted for 25% of severe dengue cases from 1980 to 2020 in the American Region.^36^

Dengue mortality is often reported as the CFR. However, severe cases are infrequent, with only 5–20% of dengue cases progressing to severe disease, causing significant morbidity and mortality.^37^ This method of reporting can underestimate the true severity of dengue as hospitalized patients face a real risk of death because of disease progression. In this study, we reported the HFR (deaths/hospitalized). Our data showed 3.25-fold increase in HFR from 0.8% in 2010 to 2.6% in 2020, with a notable rise in 2015 after changes in the reporting system associated with the introduction of the 2009 WHO dengue case classification. Although this change improved dengue case detection,^38^ it also underscores the challenges of providing timely care to individuals living in regions with limited access to health care facilities. Historically, dengue lethality in Mexico has fluctuated, with rates of 6.4% between 1980 and 1989, 2.4% from 1990 to 1999, and 0.7% between 2000 and 2007. These fluctuations are linked to multiple factors, including the introduction, circulation, and evolution of dengue serotypes and genotypes and management; the national dengue program includes training covering triage in diagnosis and management of dengue emphasizing fluid management considering the Organización Panamericana de Salud algorithms.^39^^–^^42^

In the period analyzed, the most frequently reported serotypes in Mexico were DENV-1 and DENV-2, although all four serotypes circulate in states such as Baja California Sur, Sinaloa, Nuevo León, Veracruz, Tabasco, Campeche, Yucatán, Quintana Roo, and Chiapas.^43^ This situation likely contributes to increased severe disease and hospitalization rates because of a higher probability of secondary infection.

The southern region of Mexico has the highest incidence and mortality rates for dengue. In this context, prioritizing primary care, particularly early detection; expanded health care access; control activities; health promotion; and risk communication in the southern and other endemic states is essential. Timely diagnosis and initiation of appropriate supportive care can reduce the mortality rate of severe dengue from 20% to less than 1%.^6^

Although not all dengue cases have comorbidities, 2.6% of laboratory-confirmed cases presented at least one comorbidity. Conditions such as diabetes mellitus; hematological disorders; chronic kidney disease; and liver, gastric, and autoimmune conditions can worsen severity of dengue and increase the risk of poor outcomes.^44^ In Mexico, the leading causes of death include ischemic heart disease (related to hypertension), diabetes, and liver cirrhosis.^45^ When combined with DENV infection, these conditions can increase the ORs of morbidity and mortality across all age groups.^34^ Diabetes, the second leading cause of death in Mexico, had a prevalence of 15.7% in individuals older than 20 years old in 2020, exceeding the global prevalence of 10.5%.^46^ Projections estimate an increase in diabetes prevalence to 19.7% by 2030 and 45.8% by 2045. The hallmark of severe dengue is vascular leakage, which may be exacerbated by diabetes because of endothelial dysfunction. Chronic inflammation and cytokine release in diabetes compromise vascular integrity, potentially worsening dengue outcomes.^47^ Peptic ulcer disease has a lifetime prevalence of 5–10%,^48^ and it is more common in developing countries because of higher *Helicobacter pylori *colonization rates.^49^ In regions with active DENV circulation,^1^ the coexistence of dengue-induced endothelial damage and peptic ulcers, which cause active bleeding, may increase hospitalization and mortality rates.^50^ Our findings highlight the need for tailored treatment approaches for dengue patients with comorbidities, optimizing resource allocation based on regional health care capacities.

Limited information is available regarding the impact of dengue on pregnancy.^51^^–^^54^ Our results indicate that pregnancy increases the OR for severe dengue as physiological changes during pregnancy can mask dengue warning signs, complicating diagnosis.^51^^–^^55^ Moreover, pregnant women with dengue face higher risk of hospitalization and severe disease progression, potentially leading to adverse outcomes, such as maternal mortality, stillbirth, and neonatal mortality.^56^ This issue is particularly concerning in Mexico, where the teenage pregnancy rate is high, with 70.6 births per 1,000 adolescents,^56^ coupled with a significant dengue burden in these age groups. Recent data indicate that DENV-4 poses a higher risk of severe disease in pregnant women than DENV-1 and DENV-2, particularly in hyperendemic areas where all four serotypes are present.^57^ The significance of early dengue diagnosis for pregnant women hinges on educational awareness among primary care physicians as evidenced by these data.

Previous studies have explored alternative methods to estimate dengue burden in Mexico, with different approaches and methodologies.^11^^–^^13^^,^^58^ In this context, the conditions under which costs estimations are made are important when comparing our results with the previous published data. For example, Undurraga et al.^12^ estimated costs per episode by combining patient interviews conducted in hospitals with macrocosting data from WHO estimates for Mexico and existing literature on dengue burden. They calculated indirect costs based on productivity losses by age, considering both the patient and their caregivers.^12^ Zubieta-Zavala et al.^16^ applied a microcosting approach to estimate costs per case; however, they did not explicitly account for comorbidities.^16^ It is of note that in our estimates, although the relative contribution of the comorbidities to the overall burden is lower, we incorporated this factor by calculating a weighted average that accounts for their presence.

Between 2012 and 2016,^12^ the average annual direct medical cost of dengue to the Mexican health care system was estimated at U.S. $140 million, which is U.S. $30 million higher than our 2019 estimate. However, epidemic size is not a directly comparable variable across years as the results are not linearly dependent. Instead, costs are strongly influenced by hospitalization rates, which themselves vary according to circulating serotypes and other unpredictable facts.

In other Latin American countries, the estimated cost per dengue patient was U.S. $563 in Colombia and U.S. $531.63 in Brazil.^59^ In a study by Shepard et al.,^60^ the global and regional burdens of dengue were assessed using dengue incidence estimates from the Institute for Health Metrics and Evaluation’s Global Burden of Disease Study 2013 along with multiple additional data sources. They reported that the average global cost per dengue case was U.S. $333 (range: $283–$403) for hospitalized cases and U.S. $60 ($54–$68) for ambulatory cases. Specifically for Latin America and the Caribbean, the estimated costs were U.S. $1,000 ($773–$1,251) for hospitalized cases and U.S. $102 ($88–$120) for ambulatory cases.^60^ Because of methodological differences in cost estimation, comparisons with our results should be interpreted with caution.

This study could not disaggregate the OR by age group, limiting our ability to clarify the impact of comorbidities on younger age groups, particularly those ages 5–19 years old who exhibited the highest incidence. Understanding the effects of comorbidities on severity, hospitalization rates, and mortality in these age groups in a multivariant analysis that includes age, region, and other factors together remains a key future research question.

In this study, we estimate the economic burdens of prepandemic, pandemic, and postpandemic episodes, which help us to compare the relative size of the cost that has to be spent by the public sector, and we manage to project the epidemics and costs distributions of a well-characterized sample of patients to the total dengue cases observed in our time period of analysis. This is an important contribution in that this retrospective observational study analysis uses data obtained from passive surveillance systems, which may not consistently capture all clinical cases. The databases might not be able to retrieve disease information for certain regions of Mexico where data may be unavailable without being able to distinguish the kind of urbanization between regions.

In our dataset, inherent biases include underreporting or underestimation, discrepancies in case definitions (we used ICD-10 codes), and secular trends or disease outbreaks that could potentially amplify the disease burden during specific periods. Additionally, the study did not account for the caregiver cost burden because it is not customary in Mexico to include such services. Another important component of the indirect costs, the out-of-pocket costs, was not considered in the objectives of this work, but it is worth to say that in Mexico, they are high because of repeat visits to pharmacies, travel expenses, and food among others. The cost of the government dengue prevention and control efforts was also not considered. Moreover, we were unable to calculate the age-dependent ORs of hospitalization and death, limiting our ability to analyze their implications for the dengue burden.

## CONCLUSION

Between 2010 and 2020, SINAVE recorded a total of 1,620,872 reported cases, among which 336,991 were laboratory-confirmed cases, 110,437 were hospitalizations, and 1,385 were deaths. The HFR rose from 0.8% to 2.6% in this period. Severe dengue and related deaths are no longer primarily occurring among adults but have shifted to affecting children and adolescents ages 5–19 YoA.

Comorbidities, including peptic ulcer disease, diabetes, liver cirrhosis, hypertension, kidney disease, and hematological disorders, significantly increased the risk of hospitalization and mortality in both severe and nonsevere dengue cases. During pregnancy, the risk of developing severe dengue increases. The relationship between an increase in HFR among younger individuals and comorbidities or secondary infections is unclear because of insufficient research.

Preventing dengue remains a crucial public health concern, targeting mosquito population control and surveillance of *Aedes* spp. These measures are not fully effective, and the public health authorities need to improve early dengue case detection by training and alerting health workers, identifying high-risk groups, providing appropriate clinical management, and ensuring follow-up care to prevent fatalities.

## Supplemental Materials

10.4269/ajtmh.24-0777 Supplemental Materials
