# Supplementary material for: Epidemiological and Economic Burden of Dengue in Mexico: Data Analysis from 2010 to 2020
Source: Am J Trop Med Hyg. 2025 Oct 7;113(6):1363–74. doi: 10.4269/ajtmh.24-0777 (PMC12676618; doi:10.4269/ajtmh.24-0777)
Supplement: Supplemental Materials [file tpmd240777.SD1.pdf]

Supplemental Material

This appendix has been provided by the authors to give readers additional information about their work.

Supplement to:

Epidemiological and economic burden of dengue in Mexico:

Data analysis from 2010 to 2020

Supplementary Tables Index

Table S1. Probable cases (SUIVE) due to dengue in the national healthcare system by severity (Mexico 2010 - 2020).. 4

Table S2. Probable cases (SUIVE) due to dengue in the national healthcare system by gender (Mexico 2010 - 2020).. 5

Table S3. Probable cases (SUIVE) due to dengue in the national healthcare system by region (Mexico 2010 - 2020).. 6

Table S4. Laboratory-confirmed cases due to dengue in the national healthcare system by severity (Mexico 2010 - 2020). 7

Table S5. Laboratory-confirmed cases due to dengue in the national healthcare system by gender (Mexico 2010 - 2020). 8

Table S6. Laboratory-confirmed cases by severity and deaths due to dengue in the national healthcare system by age group (Mexico 2010 - 2020). 9

Table S7. Probable and laboratory-confirmed) due to dengue in the national healthcare system by state (Mexico 2010 - 2020). 10

Table S8. Laboratory-confirmed cases due to dengue in the national healthcare system by region (Mexico 2010 - 2020). 11

Table S9. Laboratory-confirmed cases due to dengue in the national healthcare system by type of care (Mexico 2010 - 2020). 12

Table S10. Deaths in laboratory-confirmed cases due to dengue in the national healthcare system (Mexico 2010 - 2020). 13

Table S11. Deaths in laboratory-confirmed cases due to dengue in the national healthcare system by gender and type of care (Mexico 2010 - 2020). 14

Table S12. Laboratory confirmed cases due to dengue in the national healthcare system by comorbidities or other health condition (Mexico 2010 - 2020). 15

Table S13. Average length of stay (LOS) in hospitalized cases due to dengue in the national healthcare system by severity and age groups (Mexico 2010 - 2020). 16

Table S14. Average length of stay (LOS) in hospitalized cases due to dengue with comorbidities or other health condition in the national healthcare system by severity and age groups (Mexico 2010 - 2020). 17

Table S15. Average length of stay (LOS) in hospitalized cases due to dengue with complications in the national healthcare system by severity and age groups (Mexico 2010 - 2020). 18

Table S16. Estimated cost per capita of patients with dengue (public and private sector) in 2018, 2019 and 2020. 19

Table S17. Estimated direct costs in 2018 in probable cases (SUIVE) by severity and total dengue cases..... 20

Table S18. Estimated direct costs in 2019 in probable cases (SUIVE) by severity and total dengue cases..... 21

Table S19. Estimated direct costs in 2020 in probable cases (SUIVE) by severity and total dengue cases..... 22

Table S20. Estimated direct costs in 2018 to 2020 in probable cases (SINAVE). 23

Table S21. Estimated indirect costs of patients with dengue in 2018, 2019 and 2020.................................... 24

Table S22. Estimated indirect costs in 2018 to 2020 in probable cases (SINAVE). 26

Table S1. Probable cases (SUIVE) due to dengue in the national healthcare system by severity (Mexico 2010 - 2020).

Year	Non-severe dengue		Severe dengue		Total
	Cases	(%)	Cases	(%)	
2010	109,836	90.2%	11,877	9.8%	121,713
2011	15,424	70.6%	6,423	29.4%	21,847
2012	65,892	77.9%	18,719	22.1%	84,611
2013	105,973	84.2%	19,822	15.8%	125,795
2014	46,092	83.9%	8,856	16.1%	54,948
2015	61,710	91.6%	5,626	8.4%	67,336
2016	41,907	91.9%	3,717	8.1%	45,624
2017	35,413	92.4%	2,930	7.6%	38,343
2018	25,993	84.6%	4,715	15.4%	30,708
2019	142,476	90.7%	14,542	9.3%	157,018
2020	50,955	90.2%	5,512	9.8%	56,467
**2010 - 2020**	**701,671**	**87.2%**	**102,739**	**12.8%**	**804,410**

Probable cases: refers to estimated cases, which are obtained from the application of the formula (probable cases + laboratory-confirmed cases) where the probable cases are obtained by multiplying the percentage of positivity by the total number of cases without sample; **Non- Severe Dengue:** includes Dengue Fever (Classical Dengue) and Dengue without Warning Signs; **Severe Dengue:** includes Dengue Hemorrhagic Fever, Dengue with Warning Signs and Severe Dengue; **Sources:** General Directorate of Epidemiology (DGE)/Morbidity Yearbook 1984 - 2020/SUIVE/Ministry of Health/United Mexican States 2010 - 2019.

Table S2. Probable cases (SUIVE) due to dengue in the national healthcare system by gender (Mexico 2010 - 2020).

**Year**	**Female**		**Male**		**Total**
	**Cases**	**(%)**	**Cases**	**(%)**	
2010	65,065	53.5%	56,648	46.5%	121,713
2011	11,386	52.1%	10,461	47.9%	21,847
2012	45,127	53.3%	39,484	46.7%	84,611
2013	69,183	55.0%	56,612	45.0%	125,795
2014	30,518	55.5%	24,430	44.5%	54,948
2015	39,352	58.4%	27,984	41.6%	67,336
2016	26,397	57.9%	19,227	42.1%	45,624
2017	22,290	58.1%	16,053	41.9%	38,343
2018	17,026	55.4%	13,682	44.6%	30,708
2019	88,908	56.6%	68,110	43.4%	157,018
2020	30,989	54.9%	25,478	45.1%	56,467
**2010 - 2020**	**446,241**	**55.5%**	**358,169**	**44.5%**	**804,410**

Sources: General Directorate of Epidemiology (DGE)/Morbidity Yearbook 1984 - 2020/SUIVE/Ministry of Health/United Mexican States 2010 - 2019.

Table S3. Probable cases (SUIVE) due to dengue in the national healthcare system by region (Mexico 2010 - 2020).

Year	North		Center		South		Total
	Cases	(%)	Cases	(%)	Cases	(%)	
2010	30,401	25.0%	28,218	23.2%	63,094	51.8%	121,713
2011	1,149	5.3%	1,514	6.9%	19,184	87.8%	21,847
2012	8,312	9.8%	6,019	7.1%	70,280	83.1%	84,611
2013	37,164	29.5%	27,150	21.6%	61,481	48.9%	125,795
2014	28,180	51.3%	5,804	10.6%	20,964	38.2%	54,948
2015	10,598	15.7%	26,833	39.8%	29,905	44.4%	67,336
2016	8,131	17.8%	14,519	31.8%	22,974	50.4%	45,624
2017	8,722	22.7%	21,084	55.0%	8,537	22.3%	38,343
2018	4,789	15.6%	9,796	31.9%	16,123	52.5%	30,708
2019	7,736	4.9%	68,908	43.9%	80,374	51.2%	157,018
2020	16,184	28.7%	25,717	45.5%	14,566	25.8%	56,467
**2010 - 2020**	**161,366**	**20.1%**	**235,562**	**29.3%**	**407,482**	**50.7%**	**804,410**

North: Baja California, Baja California Sur, Chihuahua, Coahuila, Durango, Nuevo León, Sonora, Sinaloa, Tamaulipas y Zacatecas; **Center:** Aguascalientes, Colima, Guanajuato, Hidalgo, Jalisco, Estado de México, Michoacán, Nayarit, Querétaro, San Luis Potosí and Tlaxcala. **South:** Campeche, Chiapas, Guerrero, Morelos, Oaxaca, Puebla, Quintana Roo, Tabasco, Veracruz y Yucatán. **Sources:** General Directorate of Epidemiology (DGE)/Morbidity Yearbook 1984 - 2020/SUIVE/Ministry of Health/United Mexican States 2010 - 2019.

Table S4. Laboratory-confirmed cases due to dengue in the national healthcare system by severity (Mexico 2010 - 2020).

**Year**	**Non-severe dengue**		**Severe dengue**		**Total**
	**Cases**	**(%)**	**Cases**	**(%)**	
2010	23,599	78.3%	6,550	21.7%	30,149
2011	11,532	69.5%	5,051	30.5%	16,583
2012	33,409	64.1%	18,719	35.9%	52,128
2013	44,162	69.0%	19,822	31.0%	63,984
2014	23,760	72.8%	8,856	27.2%	32,616
2015	21,552	79.3%	5,623	20.7%	27,175
2016	14,187	79.2%	3,717	20.8%	17,904
2017	11,560	79.8%	2,930	20.2%	14,490
2018	8,625	64.7%	4,715	35.3%	13,340
2019	28,854	66.5%	14,542	33.5%	43,396
2020	19,714	78.1%	5,512	21.9%	25,226
**2010 - 2020**	**240,954**	**71.5%**	**96,037**	**28.5%**	**336,991**

Non- Severe Dengue: includes Dengue Fever (Classical Dengue) and Dengue without Warning Signs; **Severe Dengue:** includes Dengue Hemorrhagic Fever, Dengue with Warning Signs and Severe Dengue. **Sources**: National Epidemiological Surveillance System (SINAVE)/Vector-borne diseases/General Directorate of Epidemiology/Transparency Unit/INAI.

Table S5. Laboratory-confirmed cases due to dengue in the national healthcare system by gender (Mexico 2010 - 2020).

Year	Female		Male		Total
	Cases	(%)	Cases	(%)	
2010	15,725	52.2%	14,424	47.8%	30,149
2011	8,688	52.4%	7,895	47.6%	16,583
2012	27,588	52.9%	24,540	47.1%	52,128
2013	34,552	54.0%	29,432	46.0%	63,984
2014	17,858	54.8%	14,758	45.2%	32,616
2015	15,809	58.2%	11,366	41.8%	27,175
2016	10,589	59.1%	7,315	40.9%	17,904
2017	8,283	57.2%	6,207	42.8%	14,490
2018	7,297	54.7%	6,043	45.3%	13,340
2019	24,548	56.6%	18,848	43.4%	43,396
2020	13,903	55.1%	11,323	44.9%	25,226
**2010 - 2020**	**184,840**	**54.9%**	**152,151**	**45.1%**	**336,991**

Sources: National Epidemiological Surveillance System (SINAVE)/Vector-borne diseases/General Directorate of Epidemiology/Transparency Unit/INAI.

Table S6. Laboratory-confirmed cases by severity and deaths due to dengue in the national healthcare system by age group (Mexico 2010 - 2020).

Age group	Non-severe dengue		Severe dengue		Deaths	
	**Cases**	**(%)**	**Cases**	**(%)**	**Cases**	**(%)**
0 - 4	8,156	3.4%	4,487	4.7%	86	6.2%
5 - 9	17,134	7.1%	8,205	8.5%	140	10.1%
10 - 14	31,231	13.0%	11,275	11.7%	88	6.4%
15 - 19	33,095	13.7%	13,557	14.1%	93	6.7%
20 - 24	27,333	11.3%	10,530	11.0%	98	7.1%
25 - 29	23,799	9.9%	8,802	9.2%	90	6.5%
30 - 34	20,487	8.5%	7,473	7.8%	81	5.8%
35 - 39	17,708	7.3%	6,561	6.8%	92	6.6%
40 - 44	15,126	6.3%	5,809	6.0%	88	6.4%
45 - 49	13,116	5.4%	4,929	5.1%	77	5.6%
50 - 54	10,708	4.4%	4,144	4.3%	74	5.3%
55 - 59	8,073	3.4%	3,342	3.5%	59	4.3%
≥ 60	14,988	6.2%	6,923	7.2%	319	23.0%
**Total**	**240,954**	**-**	**96,037**	**-**	**1,385**	**-**

Sources: National Epidemiological Surveillance System (SINAVE)/Vector-borne diseases/General Directorate of Epidemiology/Transparency Unit/INAI.

Table S7. Probable and laboratory-confirmed) due to dengue in the national healthcare system by state (Mexico 2010 - 2020).

State	Probable cases (SUIVE)[1]		Laboratory-confirmed cases (SINAVE)[2]	
	n	(%)	n	(%)
Aguascalientes	68	0.0%	53	0.0%
Baja California	468	0.1%	303	0.1%
Baja California Sur	17,787	2.2%	11,258	3.3%
Campeche	13,545	1.7%	5,706	1.7%
Chiapas	41,387	5.1%	20,852	6.2%
Chihuahua	153	0.0%	77	0.0%
Ciudad de México	1	0.0%	1	0.0%
Coahuila	6,006	0.7%	3,418	1.0%
Colima	22,452	2.8%	7,644	2.3%
Durango	2,988	0.4%	1,703	0.5%
Guanajuato	15,676	1.9%	4,910	1.5%
Guerrero	53,455	6.6%	22,230	6.6%
Hidalgo	5,487	0.7%	3,658	1.1%
Jalisco	108,430	13.5%	32,943	9.8%
Mexico	3,026	0.4%	1,902	0.6%
Michoacán	26,976	3.4%	12,705	3.8%
Morelos	39,507	4.9%	15,138	4.5%
Nayarit	34,616	4.3%	6,478	1.9%
Nuevo León	40,422	5.0%	20,096	6.0%
Oaxaca	40,488	5.0%	13,909	4.1%
Puebla	15,692	2.0%	7,993	2.4%
Querétaro	727	0.1%	560	0.2%
Quintana Roo	30,444	3.8%	11,777	3.5%
San Luis Potosí	18,103	2.3%	7,682	2.3%
Sinaloa	21,205	2.6%	9,488	2.8%
Sonora	26,815	3.3%	11,994	3.6%
Tabasco	34,850	4.3%	14,198	4.2%
Tamaulipas	44,438	5.5%	13,885	4.1%
Tlaxcala	0	0.0%	0	0.0%
Veracruz	100,120	12.4%	52,640	15.6%
Yucatán	37,994	4.7%	21,382	6.3%
Zacatecas	1,084	0.1%	408	0.1%
**Total**	**804,410**	-	**336,991**	-

Sources: [1/]General Directorate of Epidemiology (DGE)/Morbidity Yearbook 1984 - 2020/SUIVE/Ministry of Health/United Mexican States 2010 – 2019; [2/]National Epidemiological Surveillance System (SINAVE)/Vector-borne diseases/General Directorate of Epidemiology/Transparency Unit/INAI.

Table S8. Laboratory-confirmed cases due to dengue in the national healthcare system by region (Mexico 2010 - 2020).

Year	North		Center		South		Total
	Cases	(%)	Cases	(%)	Cases	(%)	
2010	7,966	26.4%	4,163	13.8%	18,020	59.8%	30,149
2011	1,032	6.2%	1,153	7.0%	14,398	86.8%	16,583
2012	5,167	9.9%	4,079	7.8%	42,882	82.3%	52,128
2013	17,573	27.5%	11,553	18.1%	34,858	54.5%	63,984
2014	14,533	44.6%	3,804	11.7%	14,279	43.8%	32,616
2015	6,844	25.2%	7,395	27.2%	12,936	47.6%	27,175
2016	4,807	26.8%	5,098	28.5%	7,999	44.7%	17,904
2017	3,472	24.0%	6,833	47.2%	4,185	28.9%	14,490
2018	1,876	14.1%	4,180	31.3%	7,284	54.6%	13,340
2019	2,376	5.5%	17,273	39.8%	23,747	54.7%	43,396
2020	6,984	27.7%	13,005	51.6%	5,237	20.8%	25,226
**2010 - 2020**	**72,630**	**21.6%**	**78,536**	**23.3%**	**185,825**	**55.1%**	**336,991**

North: Baja California, Baja California Sur, Chihuahua, Coahuila, Durango, Nuevo León, Sonora, Sinaloa, Tamaulipas y Zacatecas; **Center:** Aguascalientes, Colima, Guanajuato, Hidalgo, Jalisco, Estado de México, Michoacán, Nayarit, Querétaro, San Luis Potosí and Tlaxcala**; South:** Campeche, Chiapas, Guerrero, Morelos, Oaxaca, Puebla, Quintana Roo, Tabasco, Veracruz y Yucatán. **Sources:** National Epidemiological Surveillance System (SINAVE)/Vector-borne diseases/General Directorate of Epidemiology/Transparency Unit/INAI.

Table S9. Laboratory-confirmed cases due to dengue in the national healthcare system by type of care (Mexico 2010 - 2020).

Year	Outpatient		Hospitalized		Total
	Cases	(%)	Cases	(%)	
2010	22,478	74.6%	7,671	25.4%	30,149
2011	9,758	58.8%	6,825	41.2%	16,583
2012	30,708	58.9%	21,420	41.1%	52,128
2013	40,335	63.0%	23,649	37.0%	63,984
2014	22,022	67.5%	10,594	32.5%	32,616
2015	20,300	74.7%	6,875	25.3%	27,175
2016	13,944	77.9%	3,960	22.1%	17,904
2017	11,241	77.6%	3,249	22.4%	14,490
2018	8,306	62.3%	5,034	37.7%	13,340
2019	28,194	65.0%	15,202	35.0%	43,396
2020	19,268	76.4%	5,958	23.6%	25,226
**2010 - 2020**	**226,554**	**67.2%**	**110,437**	**32.8%**	**336,991**

Sources: National Epidemiological Surveillance System (SINAVE)/Vector-borne diseases/General Directorate of Epidemiology/Transparency Unit/INAI.

Table S10. Deaths in laboratory-confirmed cases due to dengue in the national healthcare system (Mexico 2010 - 2020).

Year	Laboratory-confirmed cases	Deaths	Fatality Rate
2010	30,149	62	0.2%
2011	16,583	50	0.3%
2012	52,128	170	0.3%
2013	63,984	192	0.3%
2014	32,616	76	0.2%
2015	27,175	95	0.3%
2016	17,904	75	0.4%
2017	14,490	65	0.4%
2018	13,340	75	0.6%
2019	43,396	371	0.9%
2020	25,226	154	0.6%
**2010 - 2020**	**336,991**	**1,385**	**0.4%**

Sources: National Epidemiological Surveillance System (SINAVE)/Vector-borne diseases/General Directorate of Epidemiology/Transparency Unit/INAI.

Table S11. Deaths in laboratory-confirmed cases due to dengue in the national healthcare system by gender and type of care (Mexico 2010 - 2020).

Year	Gender				Type of care			
	Female	(%)	Male	(%)	Hospitalized	(%)	Outpatient	(%)
2010	32	51.6%	30	48.4%	56	90.3%	6	9.7%
2011	34	68.0%	16	32.0%	50	100.0%	0	0.0%
2012	86	50.6%	84	49.4%	161	94.7%	9	5.3%
2013	103	53.6%	89	46.4%	185	96.4%	7	3.6%
2014	44	57.9%	32	42.1%	71	93.4%	5	6.6%
2015	50	52.6%	45	47.4%	93	97.9%	2	2.1%
2016	34	45.3%	41	54.7%	74	98.7%	1	1.3%
2017	30	46.2%	35	53.8%	57	87.7%	8	12.3%
2018	44	58.7%	31	41.3%	70	93.3%	5	6.7%
2019	206	55.5%	165	44.5%	347	93.5%	24	6.5%
2020	74	48.1%	80	51.9%	131	85.1%	23	14.9%
**2010 - 2020**	**737**	**53.2%**	**648**	**46.8%**	**1,295**	**93.5%**	**90**	**6.5%**

Table S12. Laboratory confirmed cases due to dengue in the national healthcare system by comorbidities or other health condition (Mexico 2010 - 2020).

**No. of comorbidities**	**Cases**	**(%)**
One comorbidity	6,271	71.8%
Two comorbidities	2,287	26.2%
Three comorbidities	153	1.8%
Four comorbidities	20	0.2%
Total	*8,731*	-
**Comorbidities**	**Cases**	**(%)**
Diabetes	4,501	51.6%
Hemorrhagic disorders	1,143	13.1%
Hypertension	2,434	27.9%
Immunosuppression	122	1.4%
Kidney disease	112	1.3%
Liver cirrhosis	180	2.1%
Peptic ulcer disease	239	2.7%
Total	*8,731*	-
**Other health condition**	**Cases**	**(%)**
Pregnancy	5,517	100%

Note: Data not available for 2010. **Sources:** National Epidemiological Surveillance System (SINAVE)/Vector-borne Diseases/General Directorate of Epidemiology/Transparency Unit/INAI.

Table S13. Average length of stay (LOS) in hospitalized cases due to dengue in the national healthcare system by severity and age groups (Mexico 2010 - 2020).

Age Group	Non-Severe Dengue		Severe Dengue	
	Cases	Average LOS (days)	Cases	Average LOS (days)
< 1	647	3.40	1,358	4.06
1 - 4	1,035	3.10	2,569	3.57
5 - 9	2,719	3.09	6,987	3.57
10 - 14	4,138	3.16	9,028	3.30
15 - 19	4,868	3.08	10,631	3.26
20 - 24	4,124	3.00	8,199	3.19
25 - 29	3,363	2.98	6,874	3.15
30 - 34	2,673	3.01	5,849	3.30
35 - 39	2,150	3.10	5,210	3.24
40 - 44	1,666	3.09	4,555	3.23
45 - 49	1,528	3.17	3,848	3.32
50 - 54	1,373	3.33	3,309	3.38
55 - 59	1,052	3.29	2,648	3.61
60 - 64	764	3.37	1,941	3.46
65 - 69	554	3.48	1,468	3.65
70 - 74	422	3.55	993	3.61
75 - 79	312	3.65	603	4.17
≥ 80	305	3.84	674	4.17
**Total**	**33,693**	**3.14**	**76,744**	**3.36**

Sources: National Epidemiological Surveillance System (SINAVE)/Vector-borne Diseases/General Directorate of Epidemiology/Transparency Unit/INAI. Hospital Discharges/Dynamic Cubes/SAEH HEALTH SECTOR (SS, IMSS, IMSS-Bienestar, ISSSTE, PEMEX, SEDENA, SEMAR, Others). Health Statistics in Private Establishments 2010-2020/INEGI

Table S14. Average length of stay (LOS) in hospitalized cases due to dengue with comorbidities or other health condition in the national healthcare system by severity and age groups (Mexico 2010 - 2020).

Comorbidities/other health condition	Cases	Average LOS (days)
Blood disorders	250	4.78
Cardiovascular disease	469	3.93
Chronic kidney disease	54	4.56
Chronic liver diseases	33	4.73
Diabetes Mellitus	807	4.19
HIV disease	20	5.80
Neoplasms	31	4.23
Pregnancy	902	2.99
Respiratory diseases	168	4.13
Rheumatoid Arthritis	16	5.50
Other hereditary disorders	36	3.42
Others	1,287	3.78
**Total**	**4,073**	**3.82**

Sources: National Epidemiological Surveillance System (SINAVE)/Vector-borne Diseases/General Directorate of Epidemiology/Transparency Unit/INAI. Hospital Discharges/Dynamic Cubes/SAEH HEALTH SECTOR (SS, IMSS, IMSS-Bienestar, ISSSTE, PEMEX, SEDENA, SEMAR, Others). Health Statistics in Private Establishments 2010-2020/INEGI

Table S15. Average length of stay (LOS) in hospitalized cases due to dengue with complications in the national healthcare system by severity and age groups (Mexico 2010 - 2020).

Complications	Cases	Average LOS (days)
Acute liver failure	52	5.04
Acute renal failure	32	5.00
Ascites	6	6.67
Encephalopathy	2	1.50
Hepatomegaly	11	3.64
Respiratory complications	127	5.40
Sepsis	36	4.28
Others	1,622	3.61
**Total**	**1,888**	**3.81**

Sources: National Epidemiological Surveillance System (SINAVE)/Vector-borne Diseases/General Directorate of Epidemiology/Transparency Unit/INAI. Hospital Discharges/Dynamic Cubes/SAEH HEALTH SECTOR (SS, IMSS, IMSS-Bienestar, ISSSTE, PEMEX, SEDENA, SEMAR, Others). Health Statistics in Private Establishments 2010-2020/INEGI

Table S16. Estimated cost per capita of patients with dengue (public and private sector) in 2018, 2019 and 2020.

**Public Sector**	**Per capita cost ($USD)**		
	**2018**	**2019**	**2020**
Diagnosis of NSD cases	$84.41	$89.93	$90.51
Outpatient management in NSD without comorbidities	$269.27	$308.39	$318.79
Outpatient management in NSD with comorbidities	$289.21	$330.17	$340.81
Inpatient management in NSD without comorbidities	$170.99	$188.01	$193.31
Inpatient management in NSD with comorbidities	$376.76	$425.99	$440.14
Diagnosis of SD cases	$153.20	$165.26	$166.93
Outpatient management in SD without comorbidities	$271.51	$310.88	$321.52
Outpatient management in SD with comorbidities	$291.45	$332.65	$343.53
Inpatient management in SD without comorbidities	$978.20	$1,076.53	$1,097.75
Inpatient management in SD with comorbidities	$1,363.32	$1,493.09	$1,520.06
**Private Sector**	**Per capita cost ($USD)**		
	**2018**	**2019**	**2020**
Diagnosis of NSD cases	$288.82	$301.44	$311.21
Outpatient management in NSD without comorbidities	$459.62	$479.70	$495.24
Outpatient management in NSD with comorbidities	$504.11	$526.14	$543.19
Inpatient management in NSD without comorbidities	$725.3	$757.0	$781.5
Inpatient management in NSD with comorbidities	$821.2	$857.0	$884.8
Diagnosis of SD cases	$558.39	$582.80	$601.68
Outpatient management in SD without comorbidities	$476.40	$497.22	$513.33
Outpatient management in SD with comorbidities	$520.90	$543.66	$561.27
Inpatient management in SD without comorbidities	$3,927.6	$4,099.3	$4,232.1
Inpatient management in SD with comorbidities	$5,107.8	$5,330.7	$5,503.1

NSD: Non-severe dengue; **SD:** Severe dengue. Exchange rate 1 USD = 20 Mexican pesos.

Table S17. Estimated direct costs in 2018 in probable cases (SUIVE) by severity and total dengue cases.

Age group	Non-severe dengue		Severe dengue		Total	
	Total cases	Total cost ($ USD)	Total cases	Total cost ($ USD)	Total cases	Total cost ($ USD)
< 1	214	$147,462.8	121	$314,196.0	335	$461,658.8
1 - 4	840	$544,198.2	578	$1,450,304.4	1,418	$1,994,502.6
5 - 9	2,445	$1,393,057.4	1,000	$2,424,566.1	3,445	$3,817,623.5
10 - 14	3,867	$1,955,815.1	728	$1,609,961.8	4,595	$3,565,776.9
15 - 19	3,547	$1,776,803.1	506	$1,060,946.6	4,053	$2,837,749.7
20 - 24	2,830	$1,556,693.1	353	$685,998.7	3,183	$2,242,691.8
25 - 29	2,703	$1,363,248.8	317	$581,865.6	3,020	$1,945,114.4
30 - 34	2,304	$1,074,102.9	256	$491,266.1	2,560	$1,565,369.0
35 - 39	1,873	$888,515.7	202	$442,199.5	2,075	$1,330,715.2
40 - 44	1,428	$628,243.3	151	$285,036.0	1,579	$913,279.2
45 - 49	1,201	$548,457.1	143	$272,192.2	1,344	$820,649.3
50 - 54	973	$485,244.9	103	$222,258.5	1,076	$707,503.5
55 - 59	681	$344,860.9	87	$171,592.6	768	$516,453.5
60 - 64	438	$216,220.8	58	$130,082.6	496	$346,303.5
65 - 69	286	$144,011.8	45	$104,045.6	331	$248,057.4
70 - 74	141	$82,912.9	33	$71,637.2	174	$154,550.1
75 - 79	130	$91,807.3	18	$45,500.5	148	$137,307.8
≥ 80	92	$67,425.9	16	$40,858.1	108	$108,283.9
**Total**	**25,993**	**$13,309,081.7**	**4,715**	**$10,404,508.1**	**30,708**	**$23,713,589.8**

Exchange rate 1 USD = 20 Mexican pesos.

Table S18. Estimated direct costs in 2019 in probable cases (SUIVE) by severity and total dengue cases.

Age group	Non-severe dengue		Severe dengue		Total	
	Total cases	Total cost ($ USD)	Total cases	Total cost ($ USD)	Total cases	Total cost ($ USD)
< 1	1,013	$715,907.8	188	$518,913.9	1,201	$1,234,821.8
1 - 4	3,643	$2,238,315.0	593	$1,500,383.4	4,236	$3,738,698.4
5 - 9	11,563	$6,760,669.7	1,882	$4,797,812.2	13,445	$11,558,481.9
10 - 14	18,817	$10,307,930.9	2,028	$4,651,349.7	20,845	$14,959,280.5
15 - 19	18,274	$10,141,908.7	1,784	$3,895,422.8	20,058	$14,037,331.5
20 - 24	15,272	$8,722,422.3	1,386	$2,953,936.9	16,658	$11,676,359.1
25 - 29	15,031	$8,514,524.7	1,252	$2,619,594.5	16,283	$11,134,119.2
30 - 34	12,956	$6,824,967.4	1,043	$2,167,606.6	13,999	$8,992,573.9
35 - 39	10,373	$5,344,815.3	929	$2,129,193.1	11,302	$7,474,008.3
40 - 44	8,627	$4,424,023.1	757	$1,617,731.1	9,384	$6,041,754.1
45 - 49	7,961	$3,901,219.7	667	$1,445,352.7	8,628	$5,346,572.4
50 - 54	5,959	$3,173,121.2	556	$1,311,772.0	6,515	$4,484,893.2
55 - 59	4,721	$2,671,431.1	456	$1,068,611.2	5,177	$3,740,042.3
60 - 64	3,160	$1,639,718.6	321	$768,857.1	3,481	$2,408,575.8
65 - 69	2,074	$1,189,066.8	241	$642,389.0	2,315	$1,831,455.9
70 - 74	1,422	$827,460.8	177	$410,570.7	1,599	$1,238,031.5
75 - 79	780	$562,441.3	116	$312,397.7	896	$874,838.9
≥ 80	830	$604,309.8	166	$475,228.3	996	$1,079,538.1
**Total**	**142,476**	**$78,564,254.1**	**14,542**	**$33,287,122.8**	**157,018**	**$111,851,376.9**

Exchange rate 1 USD = 20 Mexican pesos.

Table S19. Estimated direct costs in 2020 in probable cases (SUIVE) by severity and total dengue cases.

Age group	Non-severe dengue		Severe dengue		Total	
	Total cases	Total cost ($ USD)	Total cases	Total cost ($ USD)	Total cases	Total cost ($ USD)
< 1	326	$244,637.3	80	$230,519.4	406	$475,156.7
1 - 4	899	$516,617.8	176	$461,548.3	1,075	$978,166.1
5 - 9	3,162	$1,849,246.8	761	$2,033,387.3	3,923	$3,882,634.1
10 - 14	5,343	$2,959,943.0	828	$2,020,779.1	6,171	$4,980,722.1
15 - 19	5,417	$2,779,567.8	568	$1,330,730.0	5,985	$4,110,297.8
20 - 24	5,901	$2,949,541.5	483	$1,077,730.9	6,384	$4,027,272.4
25 - 29	6,305	$3,117,237.5	471	$1,034,930.5	6,776	$4,152,168.0
30 - 34	5,237	$2,540,691.7	384	$853,712.3	5,621	$3,394,404.0
35 - 39	4,433	$2,229,800.4	355	$885,869.3	4,788	$3,115,669.7
40 - 44	3,699	$1,779,134.4	324	$726,584.8	4,023	$2,505,719.2
45 - 49	3,227	$1,604,594.2	294	$701,361.1	3,521	$2,305,955.3
50 - 54	2,470	$1,257,318.1	226	$561,402.5	2,696	$1,818,720.6
55 - 59	1,642	$841,922.4	169	$448,210.4	1,811	$1,290,132.8
60 - 64	1,149	$608,595.5	133	$369,634.8	1,282	$978,230.4
65 - 69	810	$432,969.5	94	$281,985.3	904	$714,954.8
70 - 74	489	$322,175.7	63	$157,388.1	552	$479,563.9
75 - 79	226	$136,647.2	52	$152,952.4	278	$289,599.7
≥ 80	220	$137,808.6	51	$143,633.3	271	$281,441.9
**Total**	**50,955**	**$26,308,449.4**	**5,512**	**$13,472,359.9**	**56,467**	**$39,780,809.3**

Exchange rate 1 USD = 20 Mexican pesos.

Table S20. Estimated direct costs in 2018 to 2020 in probable cases (SINAVE).

Year	Probable cases (SUIVE)[/1]	Total direct cost ($ USD)	Per capita cost ($USD) [/2]	Probable cases (SINAVE)[/3]	Estimated total direct cost ($ USD)[/4]
2018	30,708	$23,713,589.8	$772.2	78,621	$60,713,369.3
2019	157,018	$111,851,376.9	$712.3	268,458	$191,235,380.3
2020	56,467	$39,780,809.3	$704.5	120,639	$84,989,764.9

Exchange rate 1 USD = 20 Mexican pesos. [1/]Obtained from: Morbidity Yearbooks 1984 to 2022. SUIVE/DGE/Secretaría de Salud/United Mexican States 2010 – 2022; [2/]The annual per capita cost was calculated by dividing the total direct cost by the total number of probable SINAVE cases; [3/]Obtained from: Epidemiological Overview of Dengue 2010-2020, SINAVE/DGE/SALUD/Special Epidemiological Surveillance System for Dengue; [4/]The estimated average total direct cost was calculated by multiplying the number of probable cases (SINAVE) by the per capita cost per year.

Table S21. Estimated indirect costs of patients with dengue in 2018, 2019 and 2020.

**Indirect costs 2018**	**Probable cases (SUIVE)/1**	**Average incapacity days**	**Average incapacity days per patient**	**Total cost ($USD)**	**Average total cost per patient**
**Non-severe dengue**					
Outpatient without comorbidities	22,123	289,811	13.1	$446,231.0	$20.2
Outpatient with comorbidities	338	6,565	19.4	$4,918.4	$14.6
Inpatient without comorbidities	3,183	49,021	15.4	$61,968.7	$19.5
Inpatient with comorbidities	184	4,212	22.9	$128,075.7	$696.1
Total	*25,828*	*349,609*	*13.5*	*$641,193.7*	*$24.8*
**Severe dengue**					
Outpatient without comorbidities	758	14,723	19.4	$22,281.9	$29.4
Outpatient with comorbidities	30	583	19.4	$432.6	$14.4
Inpatient without comorbidities	3,728	85,143	22.8	$70,038.1	$18.8
Inpatient with comorbidities	146	3,338	22.9	$2,413.3	$16.5
Total	*4,662*	*103,788*	*22.3*	*$95,165.9*	*$20.4*
**Total dengue**	**30,490**	**453,397**	**14.9**	**$736,359.7**	**$24.2**
**Indirect costs 2019**	**Probable cases (SUIVE)/1**	**Average incapacity days**	**Average incapacity days per patient**	**Total cost ($USD)**	**Average total cost per patient**
**Non-severe dengue**					
Outpatient without comorbidities	119,777	1,569,079	13.1	$3,033,119.1	$25.3
Outpatient with comorbidities	3,945	76,628	19.4	$54,291.0	$13.8
Inpatient without comorbidities	17,176	264,797	15.4	$457,566.3	$26.6
Inpatient with comorbidities	662	15,061	22.8	$10,842.1	$16.4
Total	*141,560*	*1,925,564*	*13.6*	*$3,555,818.4*	*$25.1*
**Severe dengue**					
Outpatient without comorbidities	2,872	55,786	19.4	$107,107.0	$37.3
Outpatient with comorbidities	96	1,865	19.4	$1,370.2	$14.3
Inpatient without comorbidities	11,029	251,397	22.8	$401,560.8	$36.4
Inpatient with comorbidities	444	10,126	22.8	$7,257.7	$16.3
Total	*14,441*	*319,174*	*22.1*	*$517,295.8*	*$35.8*
**Total dengue**	**156,001**	**2,244,738**	**14.4**	**$4,073,114.2**	**$26.1**
**Indirect costs 2020**	**Probable cases (SUIVE)/1**	**Average incapacity days/2**	**Average incapacity days per patient/3**	**Total cost ($USD)/4**	**Average total cost per patient**
**Non-severe dengue**					
Outpatient without comorbidities	45,671	598,290	13.1	$1,975,707.0	$43.3
Outpatient with comorbidities	837	16,258	19.4	$13,681.6	$16.3
Inpatient without comorbidities	3,405	52,485	15.4	$136,273.7	$40.0
Inpatient with comorbidities	300	6,859	22.9	$5,175.0	$17.2
Total	*50,213*	*673,892*	*13.4*	*$2,130,837.3*	*$42.4*
**Severe dengue**					
Outpatient without comorbidities	949	18,433	19.4	$56,838.0	$59.9

Outpatient with comorbidities	51	991	19.4	$807.4	$15.8
Inpatient without comorbidities	4,071	92,750	22.8	$43,960.1	$10.8
Inpatient with comorbidities	406	9,304	22.9	$7,345.0	$18.1
Total	*5,477*	*121,478*	*22.2*	*$108,950.5*	*$19.9*
**Total dengue**	**55,690**	**795,371**	**14.3**	**$2,239,787.8**	**$40.2**

Exchange rate 1 USD = 20 Mexican pesos. [1/]Obtained from: Morbidity Yearbooks 1984 to 2022. SUIVE/DGE/Secretaría de Salud/United Mexican States 2010 – 2022 consider only cases in the public sector ≥15 years old; [2/]The average incapacity days were calculated by dividing the average incapacity days by the total probable cases (SUIVE); [3/]The average total cost per patient was calculated by dividing the total cost by the total probable cases (SUIVE).

Table S22. Estimated indirect costs in 2018 to 2020 in probable cases (SINAVE).

Year	Probable cases (SUIVE)[/1]	Total indirect cost ($ USD)	Per capita cost ($USD)	Probable cases (SINAVE)[/2]	Estimated total indirect cost ($ USD)[/3]
2018	30,490	$736,359.7	$24.2	49,451	$1,196,709.9
2019	156,001	$4,073,114.2	$26.1	194,195	$5,068,493.7
2020	55,690	$2,239,787.8	$40.2	93,096	$3,742,454.2

Exchange rate 1 USD = 20 Mexican pesos. [1/]Obtained from: Morbidity Yearbooks 1984 to 2022. SUIVE/DGE/Secretaría de Salud/United Mexican States 2010 – 2022, consider only cases in the public sector ≥15 years old; [2/]Obtained from: Epidemiological Overview of Dengue 2010-2020, SINAVE/DGE/SALUD/Special Epidemiological Surveillance System for Dengue, consider only cases in the public sector ≥15 years old; [3/]The estimated total indirect cost was calculated by multiplying the number of probable cases (SINAVE) by the per capita cost per year.
